# Osteopathic Palpation of the Heart

**DOI:** 10.7759/cureus.14187

**Published:** 2021-03-30

**Authors:** Bruno Bordoni, Allan R Escher

**Affiliations:** 1 Physical Medicine and Rehabilitation, Foundation Don Carlo Gnocchi, Milan, ITA; 2 Anesthesiology/Pain Medicine, H. Lee Moffitt Cancer Center and Research Institute, Tampa, USA

**Keywords:** osteopathic, visceral osteopathy, fascia, craniosacral therapy, heart

## Abstract

In the panorama of scientific literature, there is a paucity of literature on how to palpate the heart area in the osteopathic setting and relevant indications on which palpatory sensations the clinician should perceive during the evaluation. The article reviews the fascial anatomy of the heart area and the heart movements derived from magnetic resonance imaging (MRI) studies and describes the landmarks used by the cardiac surgeon to visualize the mediastinal area. The text sets out possible suggestions for a more adequate osteopathic palpatory evaluation and describes any tactile sensations arising from the patient's chest. To the knowledge of the authors, this is the first article that seeks to lay solid foundations for the improvement of osteopathic manual medicine in the cardiology field.

## Introduction and background

Visceral palpation is part of the osteopathic manipulative treatment (OMT) approach [1]. Visceral treatment can target both the abdominal area and the small pelvis, as well as the chest [2-5]. The evolution of visceral OMT has its roots in the principles of the founder of osteopathic medicine, Dr. Still, although the growth of studies and innovations in this specific field occurs much later, between the twentieth and twenty-first centuries [3,6-7]. One of the researchers and osteopaths who made an important turning point in understanding the value of the viscera in somatic symptomatology (and vice versa) was Louisa Burns, Sc.D [8]. A pillar of visceral-somatic OMT was Irvin M. Korr, Ph.D., who highlighted the importance of the wholeness of the patient [9]. Another researcher and scholar of the sympathetic and parasympathetic system, indispensable for understanding the relationships of the somatic and visceral areas, is Wilfrid Jänig, Ph.D. [10]. Jänig reminds us that we must not imagine an organ separate from the context in which it resides: "The distinct reflex patterns generated in autonomic neurons by physiological stimulation of afferent neurons innervating visceral, skin or deep somatic tissues indicate that each autonomic pathway is connected to specific neural circuits in the spinal cord, brain stem, and hypothalamus that are involved in autonomic regulation" [10]. Currently, OMT offers little emphasis on the palpation and treatment of the heart area. The best-known text in the world for studying OMT (Foundations of Osteopathic Medicine) does not leave much room for this topic, compared to other evaluation and treatment aspects in the osteopathic field (chapter 30, pages 752-753) [7]. In the collective imagination, the heart is seen as a cardiac pump that allows the cardiovascular function to act throughout the body. In reality, the myocardium has several functions, which are not only related to the fluidic field, and as we will see in detail in the continuation of the article, the heart does not work as a pump. The text reviews the functional anatomy of the heart and the anatomical area where it resides, taking into account the most recent information with the aim of proposing palpation of the heart area that is closest to updated scientific rationale. In addition, we will propose manual osteopathic approaches to the heart area, taking into account our clinical experience, trying to enrich the skills in this area with respect to the reference text for OMT.

## Review

Cardiac electrical network

**Figure 1:**
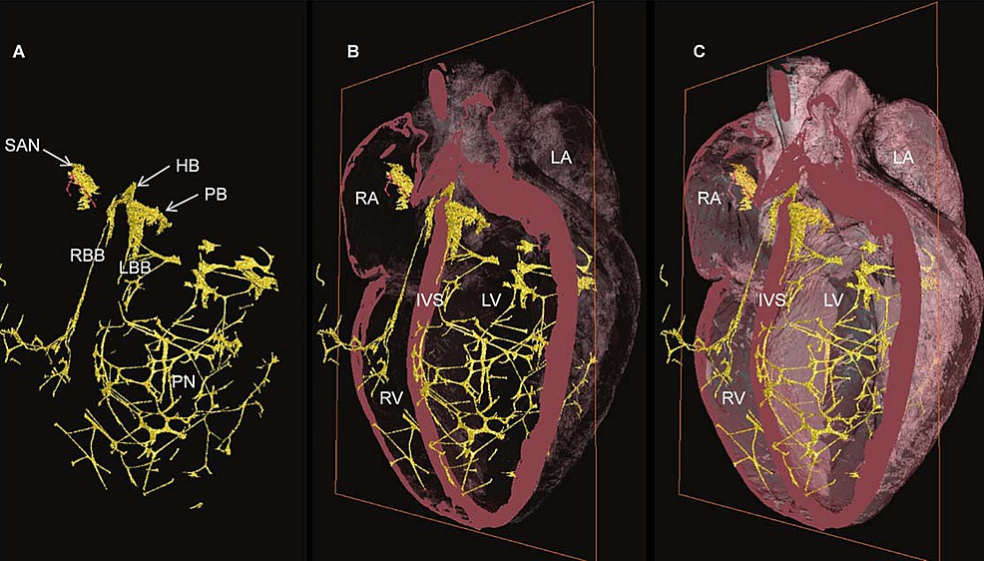
The three-dimensional image represents the electrical conduction system (CCS) of a rabbit's heart The CCS is yellow and the heart mass is pink. From A to C, we can observe the decrease in the levels of transparency of the heart, where, in Panel A, only the electrical system is highlighted. Images reproduced with the permission of Dr. Robert S. Stephenson, Comparative Medicine Lab, Department of Clinical Medicine, Aarhus University, Aarhus, Denmark. HB- His bundle, IVS- interventricular septum, LA- left atrium, LBB- left bundle branch, LV- left ventricle, PB- penetrating bundle, PN- Purkinje network, RA- right atrium, RBB- right bundle branch, RV- right ventricle, SAN- sinoatrial node, CCS- cardiac conduction system

The fundamental cells that constitute the structure and functional morphology of the heart are cardiomyocytes, acting as an entangled structure [11]. Cardiomyocytes are extraordinarily important for the electrophysiological activity of the heart, currently not fully understood [12]. Fibroblasts form the scaffolding of the heart and establish a symbiotic relationship with cardiomyocytes for the maintenance of cardiac shape-function and health [13]. We can find specialized cardiomyocytes, which develop the electrical conduction system of the heart; they are able to have spontaneous electrical activity like a pacemaker [14]. The conduction system or intrinsic electrical heart system is also known as the heart's "little brain" [15]. The intrinsic system (and the cardiac autonomic nervous system) directly affects dromotropy, inotropy, chronotropy, and cardiac lusitropy [16]. The intrinsic system is found, in particular, in the intramural ganglia and in the areas of epicardial fat, as well as in the atrial areas; this network is made up of afferent sensory pathways, ganglia that connect the whole network and sympathetic-parasympathetic neurons, the latter of which are motor-type [15-16]. The ganglia of the intrinsic system have other functions, such as endocrine and chemoreceptive; they have phasic or type I neuronal function and tonic or type II neuronal function [15]. Type II neurons contain "pacemakers" cells. Forty percent of the intrinsic neurons are contacted by the vagus nerve, although in the same intracardiac ganglion, there may be “biphenotypic” cells that respond to sympathetic and parasympathetic stimuli [15]. The intracardiac ganglia can also act without the influences of the sympathetic and parasympathetic system by extracardiac pathways, but they can be influenced by the respiratory rhythm and by nasotrigeminal receptors (trigeminal ethmoidal nerve) [15]. The extrinsic electrical conduction system of the heart can be divided into central (brainstem, forebrain, hypothalamus) and intrathoracic/extracardiac. The intrathoracic/extracardiac system includes parasympathetic (vagal) neurons, which follow intrinsic cardiac areas; the parasympathetic system carries afferents (heart and great vessels) to the medulla, sympathetic pathways, and central areas [14-16]. The right vagus nerve particularly affects the sinoatrial (SA) node while the left vagus nerve predominantly affects the atrioventricular (AV) node [16]. The parasympathetic system has a greater influence on the atrial area than the ventricular area [17]. The intrathoracic/extracardiac system also involves the sympathetic system, which projects from the C7-T1 branches that derive from the medullary mediolateral pathways or efferent preganglionic neurons; the afferent sympathetic pathways (from the heart and the great vessels) project towards the dorsal root ganglia [16]. The marriage between the sympathetic and vagal systems is very complex. The same nerve ending can release different neuropeptides for different receptors and with opposite neurochemical roles; the sympathetic system releases norepinephrine, neuropeptide Y, and adenosine triphosphate while the vagus nerve can release acetylcholine, nitric oxide, and vasoactive intestinal peptide [15]. The vagus nerve for its entire structure has sympathetic fibers, up to a maximum of about 21.53% of its volume; the types of vagal afferents (type A, B, and C) are able to stimulate the cerebral hemispheres [18]. It is not so easy to understand and identify which heart electrical system is predominant in a patient. We can recognize the conduction system as follows: the right atrial sinus node, the atrioventricular node, and His-Purkinje fibers [14]. The atrial sinus node is made up of a thin layer of cardiomyocytes capable of conducting electricity; it extends laterally with respect to the crista terminalis up to the inferior vena cava, with a maximum length of about 14.8 mm and a width of about 4.3 mm [14,19]. In its path, it can be enclosed by the epicardial fat and the neighboring myocardium; in other portions, it runs through the endocardial and epicardial areas, extending to the pectineus musculature and towards the interatrial septum [14,19]. The atrioventricular node (AV) has a ribbon-like shape at its origin while it assumes a more subtle shape towards the Purkinje network; AV originates between the coronal sinus and the membranous septum at the apex of Koch's triangle, finding its terminations around the aortic annulus [19]. The length and width of the various branches of the AV vary according to their location. The Purkinje network is more extensive in its right ventricular portion as compared to the left area; there do not appear to be intramural fibers in the human heart (Figure 1) [19,15]. The cardiac nervous system changes its behavior depending on the age of the person, with a sympathetic predominance in the elderly and children [15]. We recall that, following cardiac orthotopic transplantation, the sympathetic system regenerates after about six months while the parasympathetic system returns to involve the heart after one to three years post-surgery [15].

Organization of fibers

We do not have absolute certainty of the mode of distribution of myocytes within the heart. For some authors, it is a three-dimensional structure that resembles a torus (consisting of a single thread in the shape of a helix or spiral) or a spherical geodesic shape (differential geometry), where the cardiac contractile fibers pass through the center of this sphere [20]. The best-known model of cardiac morphological organization is that of Torrent-Guasp (1957), which shows continuity without interruption (at a macroscopic level) from the right atrium to the left ventricle, unrolling the heart during an anatomical dissection [11,20]. The latter model proposes a different myocyte distribution depending on the depth of the different heart fibers. Tractography of the heart demonstrates a helical structure but with variable myocyte vectors: in the sub-endocardial layer, the fibers are oriented longitudinally and obliquely, from the base towards the apex; in the intermediate layer, they are oriented in a circular direction while the sub-layer - epicardial is formed by the fibers of the sub-endocardial layer that re-emerge at the tip and rise with a longitudinal-oblique trend towards the base [11,14,21]. In the atrial wall, myocytes possess a longitudinal vector while in the atrial septum, we can recognize a less homogeneous distribution [14]. The orientation of the myocytes not only determines the contraction but also the electrical expression of the heart [14]. The longitudinal orientation of the myocytes, with respect to the long axis of the ventricular cavity, is called the helical angle; the myocyte radial orientation with respect to the epicardial surface is known as the intrusion angle [22]. The angle formed by the myocyte mass and the epicardial surface is called the E3 angle [22]. Sub-epicardial fibers form a counterclockwise or left-handed helix while sub-endocardial fibers form a clockwise or right-handed helix. The left ventricle has greater compactness than the right one, which is more complex and with greater trabeculae [22]. In an overall view, we could see the heart as a skein of threads while it is no longer possible to imagine the heart as a mere pump.

Heart movement

**Figure 2:**
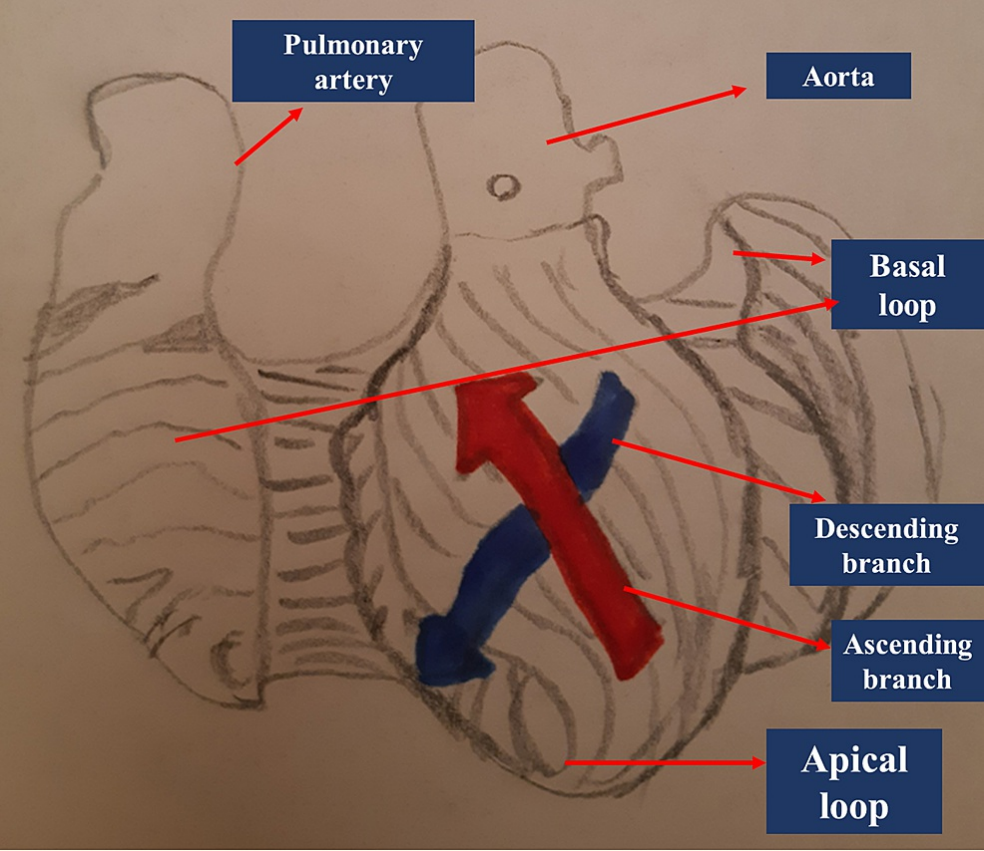
The figure shows an unrolled heart, highlighting the first outer circumferential helix and the innermost helix with the two branches, ascending and descending The image is a drawing made by Bordoni Bruno.

The heart should be considered a continuum, as muscle fibers form the heart structure without interruption; moreover, heart movement is not synchronous but sequential [11,23]. Heart movements are very complex and not fully understood. We can find six movements: uncoiling, widening, twisting, shortening, narrowing, and lengthening [23]. The most relevant action is that of twisting (or wringing), which represents the value of the ejection fraction and about 80% of the contraction mode of the right ventricle [23]. The fibers with transverse orientation and arranged in a cinconferential or helical mode (in the area referred to as the basal loop) envelop the ventricles; these fibers compress and rotate the heart [23]. In the underlying area or apical loop (forms a loop at the cardiac apex), we find a second helix conformation in which fibers intertwine, forming an angle between them of about 60 degrees; an outermost direction of these fibers is of the ascending type (from the ventricles to the atria, forming a large part of the endocardium) while the second direction of the innermost fibers is of the descending type (from the atria to the ventricles, forming a large part of the sub-endocardial area) [23-24]. This second helical formation of the heart fibers forms the septum and part of the free wall of the left ventricle; this helix allows shortening, twisting, and lengthening movements [23]. The set of these structures will produce, in addition to the movements of the different propellers and directions, the narrowing, widening, and uncoiling [23]. The first helix formation will produce, in particular, the anti-clockwise movement (before the expulsion of the blood flow), the twisting, and a return movement in a clockwise direction (post-expulsion) [23]. The first helical formation is the largest representation of the muscle mass present and covers the second helical formation [23]. The first layer or helix narrows and creates a ventricular stretching in the pre-expulsion phase or phase referred to as isovolumic; at the same time, the innermost and descending helix contracts due to ventricular shortening [23]. The shortening during expulsion stimulates torsion, with the heart base performing a clockwise rotation movement; the ascending helix stimulates the apex to perform a counterclockwise rotation movement [23-24]. The outer layer or first helix will produce a general recoil action in a clockwise direction during the post-expulsion phase, reversing its movement with respect to the pre-expulsion phase; the external ascending helix is still in a contraction phase but is brought back in a clockwise direction by the external fibers. With this mechanism of the ascending helix, ventricular elongation and the relaxation of the descending helix are created [23-24]. In theory, the branches of the internal helix (ascending and descending) should allow the flow-outflow of blood while the outermost and circumferential layer controls the return or diastole. The arrangement of the fibers allows energy savings, with maximum functional capacity while maintaining the shape of the heart. About 90 milliseconds pass between the action of the ascending and descending helix during the post-expulsion isovolumic interval [24]. The twist movement or shear deformation (the degrees of displacement) can vary according to the length of the heart and the cardiac diameter (Figure 2) [25].

Other variables that must be considered, and which affect the movement of the heart, are the movements and size of adjacent structures. In the supine position and with quiet breathing, the diaphragm muscle moves about 1.5 centimeters during an inhalation, enlarging the diameter of the rib cage by about 0.7 centimeters; with a forced inspiration, the values increase, with a diaphragmatic movement of about 7-13 centimeters and a chest diameter of about 5-11 centimeters [26]. With breathing, the heart in the absence of pathologies moves in the lateral direction (right-left) by about 1.8-6.1 millimeters, it moves in the anteroposterior direction by about 1.3-11.5 millimeters, undergoes a cranial-caudal vector movement of approximately 3.8-23.5 millimeters and a cranial-dorsal rotation of approximately 1.5-0.9 degrees [26-27]. If the space adjacent to the heart is not limited, the heart tissue can make a movement of about 11.2 mm for the base and about 6.9 mm for the apex; the heart is able to translate inwards by about 5 millimeters [26]. The heartbeat is affected and affects lung movement by approximately 1-4 millimeters; in particular, during the cardiac craniocaudal movement, the myocardium can affect the longitudinal displacement of the esophagus, of about 10 millimeters. The separation area of the diaphragm, with respect to the position of the liver and heart, is moved with the beat for about 4 millimeters [26]. In the presence of pathologies of neighboring tissues, movement should be less [26]. The same pathology involving cardiac tissue will impose morpho-functional modifications, with impaired ability to move [28]. Probably, to understand the cardiac movement dictated by the disposition of its fibers, one could trace the concepts of Riemannian differential geometry, where the geometries of curves with arbitrary dimensions are taken into consideration, knowing the measurement variables [29].

Fascial relationships of the heart

**Figure 3:**
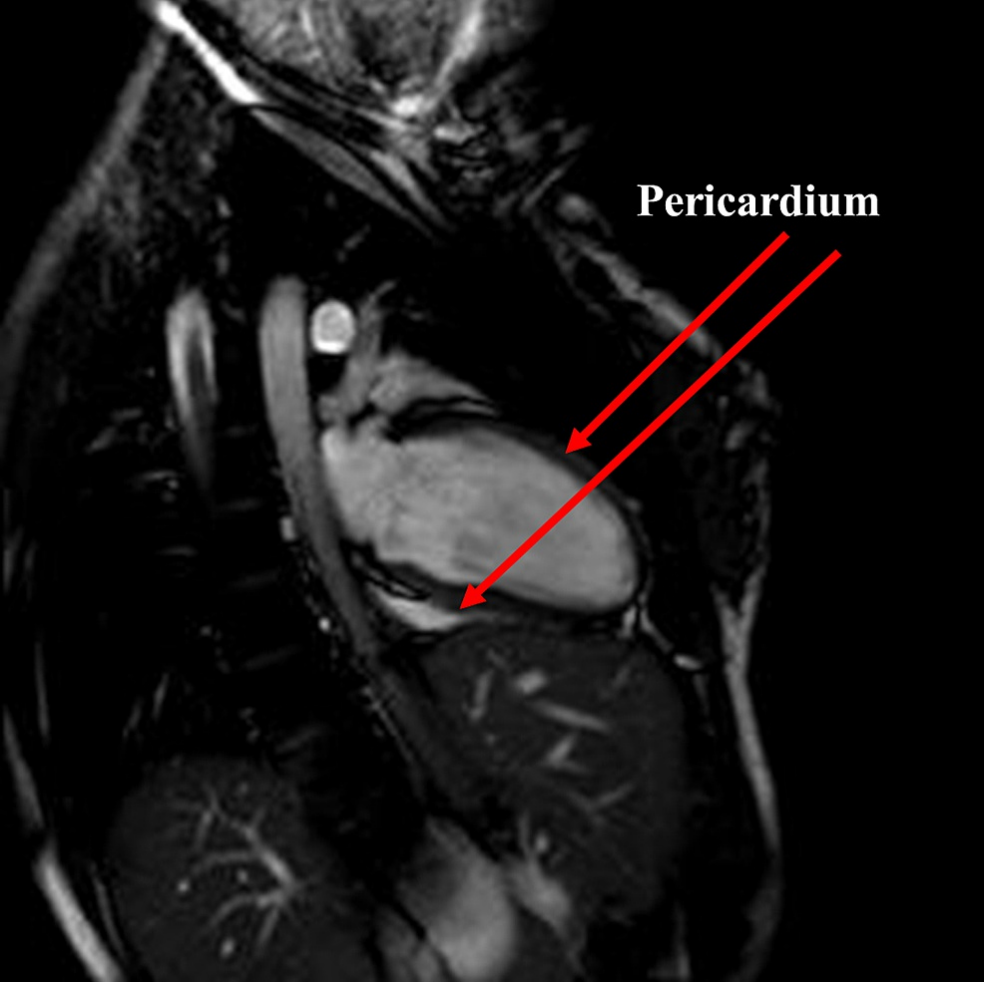
Cardiac magnetic resonance in sagittal view The arrows show the thin pericardium surrounding the heart.

All the viscera of the mediastinum are able to communicate perceived mechanical tensions, as well as their movements, through fascial bonds, which cover each viscera [30]. The heart is enveloped by the pericardium, a complex fascial structure. The pericardium has a thickness of about 1-2 millimeters, which envelops the heart and the great vessels; the fibrous pericardium on its external side is partly covered by the lungs and adipose matter [31]. The innermost layer of the fibrous pericardium is referred to as the parietal or serous pericardium. Both the pericardial layers have a high stiffness modulus, with an organization of collagenous (collagen, elastin) and wavy type fibers, which form additional layers with an orientation of about 120 degrees between them [31]. Below the parietal sheet, we find space, which contains a fluid of variable quantity (from 20 to 50 milliliters), which is closed by another pericardial layer, that is, the visceral pericardial sheet; the latter will contact the heart fat (in particular, the area of the interventricular and atrioventricular recesses, which represents about 20% of the heart's weight) and the epicardium [31-32]. The pericardium shows several recesses and sinuses. The oblique sinus (with a thickness of about 41-47 millimeters) can be found in the posterior area of the atrium and left ventricle; this sinus contacts the inferior vena cava on the right, the pulmonary veins on its left and superior portion, and, finally, its inferior area with the pericardial cavity [32]. Above the oblique sinus resides the transverse sinus formed by the anterior-medial right atrial wall and the anterior-superior wall of the left atrium; this sinus in its anterior portion is delimited by the pulmonary trunk, the ascending aorta, and the great arteries [32]. The transverse sinus comes into contact, on the left area, with Marshall's ligament and the left atrial appendage; in the upper and right areas, this sinus meets the aortocaval recess or superior sinus, which expands from the superior vena cava to the ascending aorta, coming into contact with the tracheal and paratracheal lymphatic nodes [32]. The inferior aortic recess is found in the pericardial area from the anterior medial wall of the right atrium to the aorta [32]. The retrocaval recess is formed by the pericardial folding between the superior vena cava and the right pulmonary vein while superiorly, it comes into contact with the inferior area of the right pulmonary artery [32]. Other recesses are defined as the right and left pulmonary venous recesses and are formed by the imprint of the pulmonary veins [32]. The pericardial sac rests on the connective area of the diaphragm muscle, the combination of which is called the phreno-pericardial ligament (Figure 3) [30].

The connective structures that merge with the pericardium from the sternal body (and from the IV-VI ribs) of the left side, in a postero-lateral position and forming two longitudinal lines with respect to the sternal body, are the sternum-pericardial ligaments; rarely can other ligamentous structures from the sternum (manubrium or xiphoid process) be found leading to the pericardium [30,33]. These ligaments, once they fuse with the pericardium, continue on their path; they envelop the pericardium inferolaterally, and with direction toward the thoracic vertebrae, laterally envelop the visceral structures that cross the diaphragm such as the esophagus and the descending aorta. The area of the thoracic vertebrae involved is T10-T12 and are called vertebro-pericardial ligaments [33]. There are no ligament structures that keep the pericardium suspended within the mediastinum. We can recognize other connective ligaments that connect the pericardium anteriorly (area of the left ventricle) with the bronchi and the trachea such as the tracheobronchopericardial ligaments [30]. A thin veil coming from the fascial continuum of the neck and from the thyroid cartilage, called the pretracheal fascia, covers the trachea anteriorly until it abuts the posterior portion of the pericardial sac, to merge with the connective area of the diaphragm [34]. Other fascial connections are found between the visceral fascia that covers the lungs and pericardium and the meso-esophageal fascia (esophagus-ascending aorta-pericardium) in the portion where we can find the left atrium [30]. The pericardium participates in cardiac functional expression from a mechanical and immune point of view [31,33]. The pericardial fluid is not distributed equally within the pericardial surface but is found more at the levels of the pericardial recesses and sinuses [31,33]. In a physiological context, the pericardial fluid varies its quantity during the heartbeat, whereas, normally, we can find less than 0.5 mm of fluid between the serous and visceral leaflet [31]. The presence of a healthy pericardium improves cardiac performance. The passive pressures imposed by the pericardium act as a diastolic constraint through a compressive mechanical stimulus of the radial type [31]. The cardiac apex is not bound to the pericardial visceral sheet while the base is more bound by the presence of the great vessels [31,35]. The fascial system that joins the pericardium and vice versa creates a direction of the neighboring tissues towards the heart with each systolic action and release [31].

Landmark points of the cardiac area

**Figure 4:**
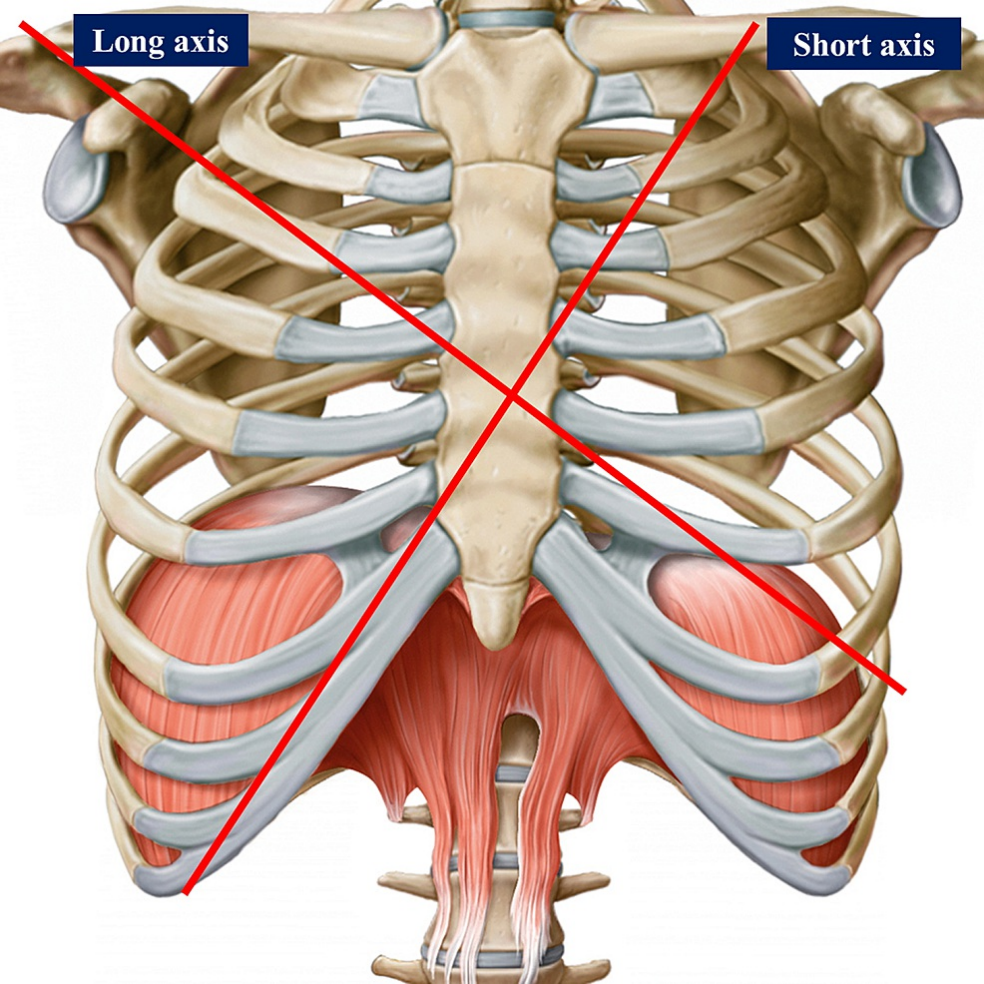
The figure highlights the drawing of a chest (bones) This drawing is given by the Edi-Ermes Publishing House, thanks to Dr. Ela Martinoli (owner of the same publishing house). The figure reworked by Bordoni Bruno illustrates the long axis and the short axis to delimit the right and left portions of the heart and to delimit the atria from the ventricles, respectively.

**Figure 5:**
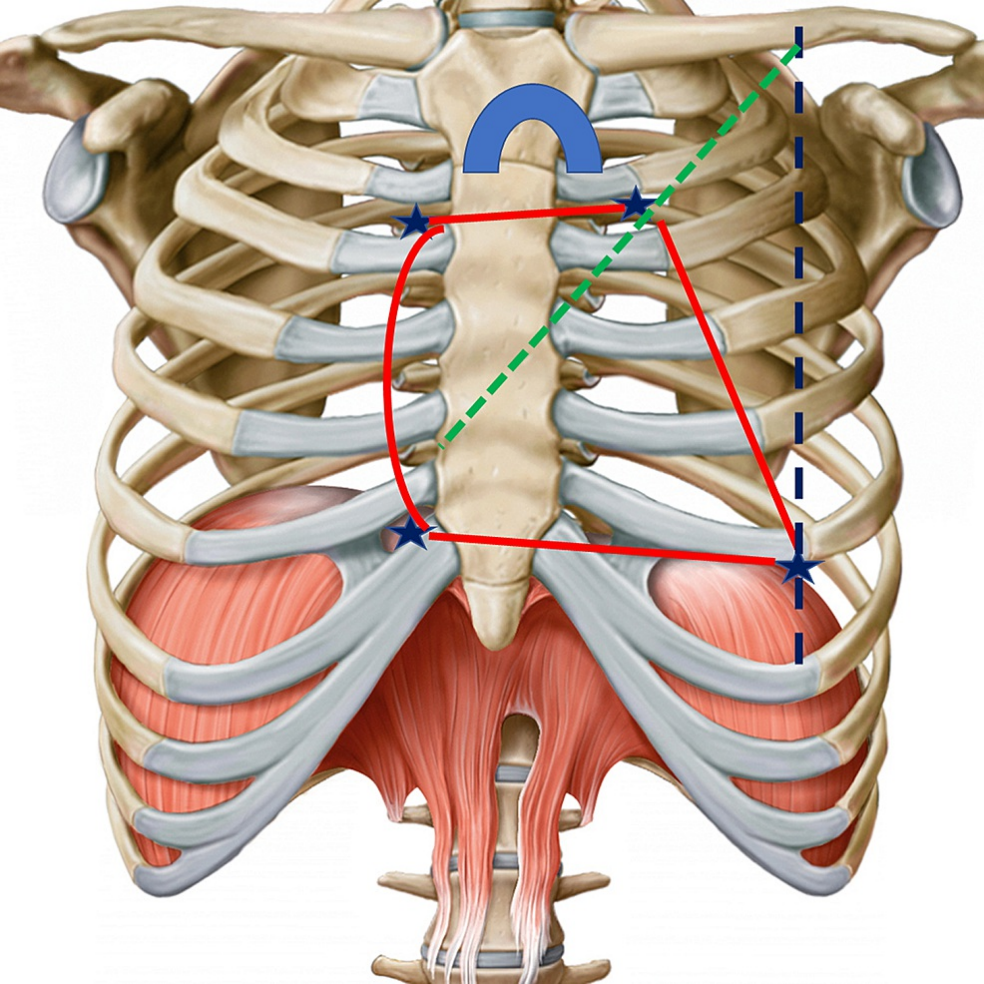
The figure highlights the drawing of a chest (bones) This drawing is given by the Edi-Ermes Publishing House, thanks to Dr. Ela Martinoli (owner of the same publishing house). The figure reworked by Bordoni Bruno illustrates four stars that delimit a quadrilateral that contains the heart mass; the dashed green line delimits the position of the valves; the dashed blue line helps understand where the cardiac apex resides; at the level of the sternal manubrium, the position of the aortic arch in blue is visible.

Using surgical landmarks of the chest, we can draw imaginary lines. A left and oblique short axis, which starts from the left hemi-clavicle up to the right chondrocostal ramp, passing through the sixth to eighth right costal cartilage and the second and third left costal cartilage; this axis separates the ventricular areas from the atrial ones [36]. A long and oblique axis starting from the left epigastrium up to the right shoulder separates the right and left portion of the heart (Figure 4) [36]. Below the manubrium of the sternum and to a small extent under the angle of Louis, we can find the aortic arch, which derives from the ascending aorta (5-7 centimeters long and about 3 centimeters wide) [36-37]. The ascending aorta, together with the pulmonary trunk, lymphatic vessels, and heart plexus are enveloped by the pericardium; at the thoracic level, we are approximately at the level of the fourth thoracic vertebra [37]. Anteriorly, the aortic arch is crossed by the left vagus nerve and the left phrenic nerve and by cardiac sympathetic branches [37]. The cardiac mass and pericardial leaflet can be found under the lower edge of the second costal cartilage, right and left, up to the cartilaginous-costal margin of the fifth and sixth ribs, right and left [36,38]. The margins of the right side of the heart reach approximately 1-2 centimeters from the sternal body; the right atrium is found under the upper margin of the third costal cartilage, up to the chondrocostal joint of the sixth-seventh rib, for about 1-2 centimeters from the sternal body [36,38]. Above the landmark of the right atrium, beyond the second sternal cartilage and upwards, there is the superior vena cava while with an imaginary perpendicular line that continues from the right atrium, inferiorly to the seventh costal cartilage, we find the inferior vena cava [36,38]. At the lower edge of the heart and with a high percentage under the sternal body, we find the right ventricle, up to about 5-8 centimeters to the left of the sternal body, at the level of the sixth costal cartilage; beyond this area, toward the left and for a total of about 6-10 centimeters from the sternal body and between the fifth to sixth ribs, there is the cardiac apex [36-38]. From the cardiac apex, it is possible to imagine a line that reaches up to the left hemi-clavicle [36]. The left side of the heart (atrium) starts from above, for about 1.25 centimeters from the sternal body to the second sternal cartilage (lower edge); the left ventricular body is, for the most part, below the remaining costal cartilages, up to the apex [36,38]. During a deep inhalation, the cardiac apex can raise up to the fourth costal cartilage [38]. We can find the heart valves by imagining an oblique line that passes from the upper edge of the third costal cartilage on the left and partially involving the sternal body of the left side. The first valve is the pulmonary one, and continuing along this line, we find the aortic valve below and slightly to the right (below the sternal body); the mitral valve is found below the fourth costal cartilage on the left, and completely under the body of the sternum, near the fourth to fifth costal cartilage on the right, is the tricuspid valve (Figure 5) [36]. The imaginary oblique line, drawn to identify the valves, could start from the left hemi-clavicle.

Palpation of the heart area

**Figure 6:**
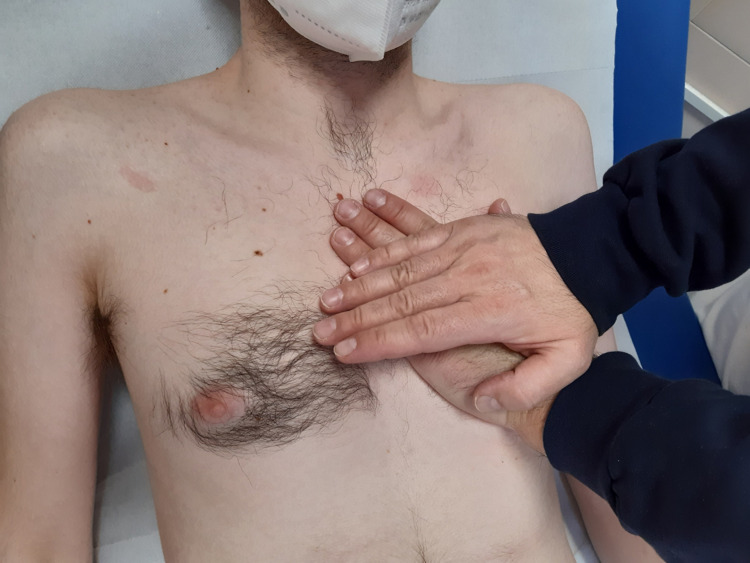
The figure illustrates the position of the osteopath's hands on the heart area with a supine patient

When we place our hands on the heart area, we must remember the presence of other structures that form the chest, such as muscles and nerve pathways. Such structures can alter the perceptual response. For example, the presence of the transversus thoracic muscle (also referred to as the sternocostalis and triangularis sterni) below the sternum and ribs anteriorly and laterally; this contractile district is very variable in size, shape, and symmetry [39]. This muscle tends to be silent in the supine position, but being covered by the endothoracic fascia, which is in close contact with the parietal pleura and part of the pericardium, it is possible to find anomalous movement variables if some adhesion or myofascial contracture is present. Furthermore, the transversus thoracic muscle can have fibers in continuity with the transverse abdominal muscle and create tractions with anomalous caudal vectors [39]. The internal intercostal muscles in contact with the parietal pleura and the endothoracic fascia, equally, can create anomalous tensions if they show structural alterations [40]. The osteopath's focus will be to discern the difference in the consistency of the different tissues that he is palpating under his hand and direct his attention to the tissue that he wishes to specifically evaluate. We must remember that all the landmarks illustrated are but a point of reference and not an absolute constraint, as we must take into account the person who is lying on the bed (age, morphological conformation of the chest and spine, pathologies). The presence of pathologies that change the cardiac morphology are capable of altering the cardiac position, as well as the presence of pulmonary or diaphragmatic pathologies (hernias or inspiratory or expiratory attitudes) [41-42]. With palpation, it is necessary to keep present the passive tensions of the pericardium and the tensions produced by the neighboring tissues in fascial connection with the pericardium; we do not have to hear the heartbeat but how the heart moves inside the pericardium and how the latter behaves inside the mediastinum. The osteopath must search the space of movement and evaluate the available entities [43]. The custom of imagining an organ moving on three planes, during the palpatory act, can be a mistake, as the organ is a three-dimensional structure and as such, it moves on all planes. The operator can be positioned next to the patient in the way the clinician prefers because it will be the tranquility and posture chosen that will help the palpatory response; equally, the patient should be positioned in a comfortable position. With experience in cardio-pneumology for almost 30 years, we recommend positioning on the left side of the patient, while he is supine. The hand rests on the chest as if to trace the underlying position of the heart mass (the listening hand), while the second hand rests on the first (pressing hand). Palpation is able to discriminate microscopic movements (tens of micrometers), and the osteopath's hand is able to discern movements of all the fascial components that he listens to with touch, solid or fluid [44]. The perceived movements are slow and large and do not reflect the real-time movements of the heart and neighboring tissues. Why? Probably, osteopathic palpation perceives the slower movements of the fascial continuum (solid and fluidic); we could speak of a perception of the fascial echo, that is, since each cell vibrates and moves, it leaves an imprint. This imprint is the pattern or patterns that make up the macroscopic movement, as the microscopic forms the structure we perceive and see. Furthermore, we must remember that any phenomenon we observe, we do not observe from the outside, but from the inside, as we are part of what we perceive and see [45-47]. We got the imprint of biotensegrity from an architectural concept [48]. In reality, we cannot understand bodily connections without considering the fluids or liquid band (fascintegrity), and all the microscopic phenomena of matter. We should speak of biointeraction, that is, extrapolating a concept of quantum physics, each body portion interacts bi-directionally [49-50] - a three-dimensionality in motion that involves aspects of the body not always taken into consideration. It is not a question of mechanism but of organicism [46-47]. What movements should the osteopath feel from the heart area? The "systolic memory" imprinted in the fascia and of the fascia should allow the palpatory perception of a clockwise (cardiac base) and counterclockwise (cardiac apex) movement, a caudal translation, an oblique dragging to the left, and inward traction of the chest; the movements are slow, harmonious, and fluid. The "diastolic memory" will produce the opposite actions, with a clockwise (cardiac apex) and counterclockwise (cardiac base) action, an oblique translation to the right side, a cranial movement, and a "push" from the chest toward the hand. The rhythm, the entity, the perceived force must be harmonious or, otherwise, palpation could reveal a problem, not necessarily related to the palpated organ. The manual pressure to be applied on the chest will be minimal and sufficient to cross the bone and muscle tissue until the heartbeat is perceived clearly; it will be a pressure that gently and gradually increases, respecting the patient's sensations. If the patient feels discomfort, both of the structures under pressure and of an emotional nature, the osteopath will stop his evaluation or will leave only the listening hand in support. If the patient is unable to stay in the supine position, the osteopath may decide to palpate the heart area with the patient sitting. The clinician will position himself on the left side of the patient (better if the clinician also has a chair to be comfortable), with the listening hand on the heart area, while the other hand will be placed parallel to the previous one, in order to allow the maintenance listening position (Figure 6).

**Figure 7:**
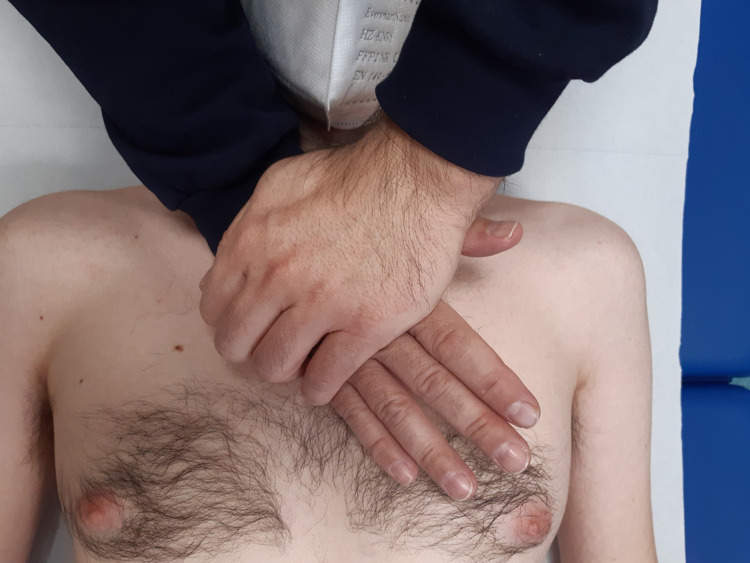
The figure illustrates how the osteopath could palpate the structure that constitutes the aortic arch and the surrounding space

For palpatory listening to the aortic arch, it is preferable for the clinician to position himself at the patient's head, with one hand listening and the other overlapping and pressing (Figure 7). He leans on the manubrium of the sternum and in a small part on the sternal body, the eminence hypotenary, while the other hand presses gently. The pressure to be applied is minimal. The goal of palpatory listening will not be to sense the blood flow but will be to able to perceive the behavior of the fascial structure that constitutes the aortic arch, as well as the available space of the adjacent structures. The osteopath should perceive with palpation cranial movement, a clockwise torsion and dragging to the left, which actions would correspond to the systolic memory. The opposite movement, such as a counterclockwise twist, a caudal vector, and a drag to the right would represent diastolic memory. The perceived movements are slow and not equivalent to the real motion of the aortic arch or the velocity of the flows. Remember that there may be congenital anomalies of the aortic arch (double aortic arch, left aortic arch, right aortic arch) and that such aberrations may be physiological; it is always better to keep in mind that a palpatory anomaly is not necessarily a sign of the presence of pathology [37]. It is the clinician who must adapt to the patient and not the contrary: this is the basis for stimulating the patient's intrinsic salutogenic response in the best possible way.

As for the palpation of the heart valves, it has no real clinical application, as the palpation of the heart mass itself can give important indications. It is not possible to improve the valve function in this manner.

## Conclusions

The article reviewed the anatomy of the heart area and the movements with which the heart expresses itself during systole and diastole. The fascial relationships involving the cardiac mass, such as the pericardium, and the fascial connections with other neighboring organs, as well as the somatic structures present, have been highlighted. To the knowledge of the authors, it is the first article in the scientific literature that describes how the palpation of the heart area should be performed and what, possibly, the osteopathic clinician should feel with palpation. Palpation of the heart area is often underestimated by the osteopathic clinician, although it falls within the concept of the law of the artery. We believe that the text can offer new insights into manual osteopathic medicine.
